# Insights into the Bidirectional Properties of the Sheep–Deer Prion Transmission Barrier

**DOI:** 10.1007/s12035-018-1443-8

**Published:** 2018-12-27

**Authors:** Chafik Harrathi, Natalia Fernández-Borges, Hasier Eraña, Saioa R. Elezgarai, Vanessa Venegas, Jorge M. Charco, Joaquín Castilla

**Affiliations:** 1 CIC bioGUNE, Parque tecnológico de Bizkaia, 48160 Derio, Bizkaia Spain; 2 IKERBASQUE, Basque Foundation for Science, 48011 Bilbao, Bizkaia Spain

**Keywords:** Prion diseases, Transmissible spongiform encephalopathy (TSE), Scrapie, Chronic wasting disease (CWD), Transmission barrier, In vitro propagation, PMCA

## Abstract

The large chronic wasting disease (CWD)-affected cervid population in the USA and Canada, and the risk of the disease being transmitted to humans through intermediate species, is a highly worrying issue that is still poorly understood. In this case, recombinant protein misfolding cyclic amplification was used to determine, in vitro, the relevance of each individual amino acid on cross-species prion transmission. Others and we have found that the β2–α2 loop is a key modulator of transmission barriers between species and markedly influences infection by sheep scrapie, bovine spongiform encephalopathy (BSE), or elk CWD. Amino acids that differentiate ovine and deer normal host prion protein (PrP^C^) and associated with structural rigidity of the loop β2–α2 (S173N, N177T) appear to confer resistance to some prion diseases. However, addition of methionine at codon 208 together with the previously described rigid loop substitutions seems to hide a key in this species barrier, as it makes sheep recombinant prion protein highly susceptible to CWD-induced misfolding. These studies indicate that interspecies prion transmission is not only governed just by the β2–α2 loop amino acid sequence but also by its interactions with the α3-helix as shown by substitution I208M. Transmissible spongiform encephalopathies, characterized by long incubation periods and spongiform changes associated with neuronal loss in the brain, have been described in several mammalian species appearing either naturally (scrapie in sheep and goats, bovine spongiform encephalopathy in cattle, chronic wasting disease in cervids, Creutzfeldt–Jakob disease in humans) or by experimental transmission studies (scrapie in mice and hamsters). Much of the pathogenesis of the prion diseases has been determined in the last 40 years, such as the etiological agent or the fact that prions occur as different strains that show distinct biological and physicochemical properties. However, there are many unanswered questions regarding the strain phenomenon and interspecies transmissibility. To assess the risk of interspecies transmission between scrapie and chronic wasting disease, an in vitro prion propagation method has been used. This technique allows to predict the amino acids preventing the transmission between sheep and deer prion diseases.

## Introduction

Transmissible spongiform encephalopathies (TSEs) or prionopathies are fatal neurodegenerative disorders affecting both humans and animals. Kuru and Creutzfeldt–Jakob disease (CJD) in humans, chronic wasting disease (CWD) in deer and elk, scrapie in sheep and goats, and bovine spongiform encephalopathy (BSE) in cattle are some of the best known TSEs. The fundamental pathogenic event in all of them is the conversion of the normal, protease-sensitive host prion protein (PrP^C^) to an abnormally folded, partially protease-resistant form (PrP^Sc^) that accumulates in the central nervous system of the affected individuals [1]. Scrapie, a naturally occurring TSE in sheep and goats, has been known for over 200 years and is endemic in many parts of the world. Despite the fact that transmission to human beings has never been reported, surveillance programs have been established in several countries since the 1990s in order to assess its prevalence and limit its spread [2]. As one of the better characterized TSE, several distinct scrapie prions have been described [3] showing slightly different three-dimensional structures and named prion strains [4]. Similarly, CWD occurs naturally in captive cervids plus it is the only known prion disease maintained in any free-ranging wildlife species including mule deer (*Odocoileus hemionus*), elk (*Cervus canadensis*), and moose (*Alces alces*). Since its initial report in the late 1960s [5], CWD has spread extensively throughout the USA, where it is present in 25 states, and 3 Canadian provinces [6]; it was present in South Korea [7] and the first cases in Europe were reported recently in a reindeer (*Rangifer tarandus*) and moose (*Alces alces*) [8, 9]. The high infectivity and rapid transmission between cervids result in a high prevalence which can exceed 90% in captive animals [10]. This consequently leads to increased exposure of humans and other animals such, as cattle and sheep, to CWD through consumption of prion-infected animal products or gazing on prion contaminated pastures, respectively. Thus, the zoonotic risk of CWD or transmission to other animal species is increasing and CWD transmissibility studies are now of great interest to public health. Unlike scrapie, only two different CWD strains have been described so far [11], although the recently described European CWD seems to be different from the previous ones [8].

The transmission of prions from one species to another is partially restricted as the ability to infect some species but not others is an intrinsic characteristic of each prion strain. This phenomenon, known as transmission barrier, usually manifests as an incomplete attack rate and a long incubation period (time from inoculation to onset of clinical signs) upon initial interspecies transmission and this typically becomes shorter for each serial passage [12]. Several experimental inoculations have been performed to study interspecies transmission of CWD, most by intracerebral (IC) inoculation. By this route, CWD has been successfully transmitted to ferrets (*Mustela putorius furo*) [13] but failed to transmit to Syrian golden hamsters (*Mesocricetus auratus*) even after multiple attempts [10]. However, after serial passages, the ferret-adapted CWD was readily transmissible to hamsters, showing that adaptation to a new host can alter interspecies transmissibility [13]. In small ruminants, IC inoculation of CWD from mule deer to goats resulted in an incubation period of about 6 years, much longer than what occurs with scrapie [10]. In addition, initial IC transmission of the CWD agent from deer to sheep of the Suffolk breed is usually associated with significantly longer incubation periods (2160 dpi) compared to scrapie (570 dpi) [14, 15]. Conversely, IC inoculation of elk with brain homogenate from scrapie-affected sheep causes a disease resembling scrapie that is indistinguishable from CWD by histologic examination or immunohistochemistry. However, there was an extended incubation period (660 dpi) [16], although not as much as when CWD was transmitted to sheep, showing that transmission barriers are not uniformly bidirectional [17]. Transmission experiments of CWD prions from white-tailed deer, mule deer, and elk to transgenic mice expressing deer, elk, sheep, cattle, or human PrP^C^ suggest that the transmission barrier for CWD prions among different species of the cervid family is low, whereas the transmission barrier for CWD prions to sheep, cattle, and humans is high [18–22]. In contrast, scrapie and sheep-passaged BSE prions are readily transmitted to transgenic mice expressing elk PrP [23, 24]. However, besides the initially described CWD strain, few other have arisen as the disease spread and was further studied, being their transmission properties different from that most studied and described in the previous lines [25, 26].

Previous studies have established that the PrP amino acid sequence strongly affects both PrP^Sc^ formation and interspecies transmission of the TSE agent [17, 27–29]. Furthermore, in vitro studies using conversion systems based on the incubation of prions with PrP from diverse sources have facilitated studies of the species barrier and identified critical PrP residues that control prion transmission [30, 31]. These in vitro methodologies are one of the preferred ways to study the molecular determinants of the species barrier even though they differ from natural transmission. The protein misfolding cyclic amplification (PMCA) technique [32, 33] is one such powerful in vitro tool that mimics the in vivo conversion process of PrP^C^ to PrP^Sc^ but with accelerated kinetics. It amplifies minute quantities of any PrP^Sc^ present in a sample [34] and has been critical in studies of the transmission barrier and strain phenomenon of prions [35, 36]. Using a variant of this technique (recombinant PMCA, rec-PMCA), in which recombinant PrP (rec-PrP) is used as source of PrP instead of brain homogenates [37–39], we aim to decipher the determinants of the sheep–deer transmission barrier as proof of concept for the rec-PMCA as a tool by which we can determine the relevance of each amino acid residue in prion proteins that could be involved in a particular species barrier.

Using this in vitro technique, we wanted to investigate the molecular determinants of the transmission barrier of a cervine CWD strain to ovine and their effects on the transmissibility of ovine prions. Several sheep prion strains and an elk CWD prion strain were propagated in substrates containing sheep and mule deer rec-PrP, respectively. There is one amino acid difference between elk and mule deer PrPs and mule deer rec-PrP has previously shown much better in vitro conversion efficiency in our hands. Therefore, despite using a CWD strain from elk, mule deer rec-PrP was used as a substrate. These misfolded rec-PrPs were then used to test their ability to propagate in vitro to substrates with sheep rec-PrPs with different mule deer substitutions. Through the different propagation abilities shown by the mutant ovine rec-PrPs when seeded with distinct sheep or mule deer misfolded rec-PrP, the effect of each substitution on the transmission barrier was analyzed. Therefore, we developed a rapid method to perform a preliminary evaluation of transmission barriers, based on in vitro propagation of protease-resistant misfolded recombinant PrP with different substitutions. Our results, consistent with several other previous studies on this interspecies transmission barrier, show that besides the β2–α2 loop amino acid sequence, its interactions with α3-helix might be critical to the transmission of CWD to sheep. Moreover, they indicate that rec-PMCA may be one of the most useful tools available for reliable and cost-effective studies for the detection of residues and regions critical to interspecies prion transmission.

## Materials and Methods

### Cloning and Recombinant PrP Expression

For cloning of wild-type ovine PrP (sheep ARQ) PCR was used to amplify the coding sequence of residues 23–231 using primers ov1 *5′-AGGAGATATACCATGAAGA AGCGACCAAAACCTGG-3′* and ov2 *5′-GTGATGGTGATGTTAACTTGCCCCCCTTTGGTAATA-3′* (all primers synthesized by Sigma-Aldrich). As template, we used genomic DNA from sheep homozygous for alleles 136A, 154R, and 171Q and the open reading frame (ORF) of the ovine *PRNP* gene was cloned into pOPIN E vector (Oxford Protein Production Facility UK). All amino acid substitutions between ovine and mule deer PrP were carried out by two-step PCR initially using internal primers for the particular substitution and then external primers ov1/ov2 for the amplification of the mutant template. The S98T mutation was introduced by forward primer (FW) *5′-GGTCAAGGTGGTACCCACAGTCAGTGG-3′* and reverse primer (RV) *5′-CCACTGACTGTGGGTACCACCTTGACC-3′*, the S173N mutation with FW *5′-CCAGTGGATCAGTATAATAACCAGAACAAC-3′* and RV *5′-GTTGTTCTGGTTATTATACTGATCCACTGG-3′* primer, the N177T with FW *5′-GTAACCAGAACACCTTTGTGCATG-3′* and RV *5′-CATGCACAAAGGTGTTCTGGTTAC-3′*, and the I208M substitution with FW *5′-GACATCAAGATGATGGAGCGAGTGG-3′* and RV *5′-CCACTCGCTCCATCATCTTGATGTC-3′*. Therefore, multiple mutations were generated by the substitution of amino acids at residues 98, 173, and 208 using ovine N177T as a template. The constructions containing the double mutants S98T, N177T and I208M, N177T were generated by using, respectively, the plasmids described above (S98T and I208M rec-PrP). S98T and I208M plasmids were digested with XbaI-PstI and PsyI-BspHI (all restriction enzymes were from Thermo), respectively; then the 589- and 330-bp fragments were ligated into XbaI-PstI or PsyI-BspHI double-digested pOPIN E vector containing ovine N177T rec-PrP. The S173N/N177T ovine multiple mutants were made by two-step PCR using the internal primer pairs: FW *5'-CCAGTGGATCAGTATAATAACCAGAACACCTTTGTGCATGACTGTG-3′* and RV *5′-CACAGTCATGCACAAAGGTGTTCTGGTTATTATACTGATCCACTGG-3′* and then external primers ov1/ov2 for the amplification of the mutant template. The S98T/S173N/N177T and I208M/S173N/N177T were generated by digestion of S98T and I208M plasmids with XbaI-PstI and PsyI-BspHI, respectively; then the 589- and 330-bp fragments were ligated into XbaI-PstI or PsyI-BspHI double-digested pOPIN E vector containing ovine S173N/N177T rec-PrP. The sequences of all plasmid constructs were confirmed by sequencing with T7 oligonucleotide 5′-TAATACGACTCACTATAGGG-3′ using the Stabvida (Lisbon, Portugal) sequencing service.

Rosetta (DE3) *Escherichia coli* were used for expression of ovine recombinant rec-PrPs. Proteins were produced as described previously [37, 38, 40]. Briefly, *E. coli* were cultured in Luria–Bertani medium (LB) with ampicillin (20 μg/ml) (Sigma-Aldrich) to an optical density between 0.6 and 0.8 at 600 nm and PrP expression was induced for 3 h by addition of isopropyl β-D-1-thiogalactopyranoside (IPTG) (Gold Biotechnology) to a final concentration of 1 mM. Cells were harvested and cooled in ice for 15 min before centrifugation at 4,500*g* for 15 min at 4 °C. Cells were lysed for 30 min at room temperature by shaking (200 rpm) in lysis buffer (50 mM Tris HCl (Fisher Bioreagents), 5 mM EDTA (Sigma-Aldrich), 1 mM phenylmethylsulfonyl fluoride (PMSF) (Sigma-Aldrich), 100 μg/ml lysozyme (Sigma-Aldrich), and 1% Triton X-100 (Amresco); pH 8) in the presence of DNase I (100 UI/ml) (Sigma-Aldrich) and MgCl_2_ (20 mM). The lysate was centrifuged at 8,500*g* for 1 h. After centrifugation, the pellet was washed by thorough resuspension in 50 ml washing buffer (20 mM Tris HCl, 150 mM NaCl (Sigma-Aldrich), 1 mM EDTA (Sigma-Aldrich), 1% Sarcosyl (Sigma-Aldrich) at pH 8.0) and centrifuged again at 8,500*g* for 30 min. The pellet was resuspended in 6 ml of inclusion buffer (20 mM Tris HCl, 0.5 M NaCl (Sigma-Aldrich) and 6 M guanidine HCl (Fisher Scientific) at pH 8.0) and incubated at 37 °C overnight. After centrifugation at 8,500*g* for 1 h at 4 °C, the supernatant was filtered through a 0.20-mm PVDF syringe filter (Minisart, Sartorius Stedim).

The purification was based on the use of a histidine affinity column (*HisTrap FF Crude 5 ml*, GE Healthcare Amersham). The column was assembled on the pump system (Masterflex Peristaltic Pumps), washed, and then filled with binding buffer (20 mM Tris HCl, 500 mM NaCl, 5 mM Imidazole (Sigma-Aldrich), 2 M guanidine HCl at pH 8). After loading the sample into the column, the protein was eluted using elution buffer (20 mM Tris HCl, 500 mM NaCl, 500 mM Imidazole, 2 M guanidine HCl at pH 8). The eluted sample (30 ml) was loaded as a denatured protein in the guanidine HCl solution (6 M), followed by concentration to 4–5 mg/ml using 10-kDa *centrifugal filter* (Amicon Ultra-15 10 kDa unit, Millipore) and the purified protein was stored at − 80 °C. For all proteins used in this study, the purity was confirmed by Coomassie-stained 4–15% polyacrylamide gels (Bio-Rad).

### Preparation of PMCA Substrates

Brains from PrP knout out (*PRNP*^*−/−*^) mice [41] or chicken were homogenized to a 10% solution in conversion buffer (CB) (1% Triton X-100 and 150 mM NaCl in phosphate-buffered saline (PBS) (Fisher) plus complete protease inhibitors cocktail (Roche)). Homogenates were cleared by centrifuging at 19,000*g* for 1 h at 4 °C. At the same time, ovine rec-PrP, purified as described above, was diluted 1/5–1/10 in PBS and refolded by dialysis against PBS buffer for 1 h at 4 °C using a Slide-A-Lyzer Dialysis Cassette (Thermo Scientific).

After centrifugation at 19,000*g* for 15 min at 4 °C, the soluble rec-PrP was mixed at 1:10 proportion with the brain homogenates described above. The concentration of ovine rec-PrP was measured by its absorbance at 280 nm and confirmed by bicinchoninic acid (BCA) assay (Thermo-Pierce). The PMCA substrates were aliquoted in 200 μl PCR tubes and stored at − 80 °C until required.

### Prion Strains

A panel of TSE agents, including cattle BSE (Laboratorio Central de Veterinaria, Madrid, Spain), elk CWD (University of California San Diego, San Diego, USA), and atypical BSE (BSE-H and BSE-L), and different sheep scrapie strains (Dawson (French National Institute for Agricultural Research, Toulouse, France) Langlade, UKA2 and SC21 (Centro de Investigación en Sanidad Animal, Madrid, Spain)) (Table 1) were prepared from infected brain tissues as 10% (*w*/*v*) homogenates in 5% glucose. The homogenate stock was aliquot and stored at − 80 °C.

**Table 1:** Description of the different prions used as seeds

Name	Origin	Description	Supplier
Dawson (Pg127)	France	Classical scrapie from a naturally infected sheep	(INRA^T^, France)^a^
Langlade	France	Classical scrapie from a naturally infected sheep	(CISA^M^, Spain)^b^
UKA2 (19KD)	UK	Classical scrapie from a naturally infected goat	(CISA^M^, Spain)^b^
SC 21 (21KD)	France	Classical scrapie from a naturally infected sheep	(INRA^T^, France)^a^
BSE	Spain	BSE from a naturally infected cow	(LCV^M^, Spain)^c^
BSE-H	France	BSE from a naturally infected cow	(LCV^M^, France)^c^
BSE-L	France	BSE from a naturally infected cow	(LCV^M^, France)^c^
CWD	USA	CWD from a naturally infected elk	(UCSD^C^, USA)^d^

^a^French National Institute for Agricultural Research (INRA), Toulouse, France

^b^Centro de Investigación en Sanidad Animal (CISA), Madrid, Spain

^c^Laboratorio Central de Veterinaria (LCV), Madrid, Spain

^d^University of California San Diego (UCSD), CA, USA

### Protein Misfolding Cyclic Amplification

PMCA was performed as described by Castilla et al. [33, 34, 42]. Briefly, PMCA samples were added to a reaction mixture that was prepared by adding 5 μl of PrP^Sc^ inoculum to 45 μl of PMCA substrate prepared as described above. The mixture was loaded into 0.2 ml PCR tubes (Fisherbrand), positioned on an adaptor placed on the plate holder of a microsonicator (Misonix Q-700, QSonica), and subjected to PMCA, each consisting of 30 min incubation at 38 °C followed by a 15-s pulse of sonication set at 50–60% amplitude. The conversion efficiency was determined in a single 48 h rec-PMCA round with 1 mm diameter zirconium-silica beads (BioSpec Products) using serial dilution of the initial inoculum. For the ovine rec-PrP^res^ generation, serial rounds of 24 h PMCA were performed. Each new round was seeded at a 1:10 dilution with the product of the previous PMCA round for 24 rounds. To avoid any cross-contamination, *PMCA* reaction tubes were sealed with *parafilm* (Bemis). Non-amplified control aliquots were always taken and frozen at − 80 °C to be used as a reference as initial seed content. In all the experiments, four replicates of each inoculum were used and four replicates of negative controls, each comprised of 50 μl of unseeded substrate, were included.

### Western Blot

The PMCA products were digested with 25 μg/ml of Proteinase K (PK) (Roche) in the presence of 10% sarkosyl in PBS for 1 h at 42 °C with continuous agitation. Digestion was stopped by adding 10 μl of sample buffer *NuPAGE* (Invitrogen Life Technologies) and by heating to 100 °C for 10 min. Samples were run on Criterion 4–15% acrylamide gels (Criterion TGX gel, Bio-Rad) and transferred to polyvinylidene difluoride (PVDF) membranes (Trans-Blot Turbo Transfer Pack PVDF, Bio-Rad). For the immunoblotting experiments, mouse monoclonal antibody 9A2 (Central Veterinary Institute) was used at concentrations of 1 μg/ml. Rec-PrP^res^ signal development was performed with horseradish peroxidase-conjugated goat anti-mouse immunoglobulin G (*IgG-HRP*, Santa Cruz Biotechnology). Immunoreactivity was detected using SuperSignal West Pico chemiluminescent system (Thermo Scientific Pierce), visualized, and quantified using the *FluorChem Q* system and *AlphaView Q* software (Alpha Innotech) [37].

## Results

### In Vitro Generation of Misfolded Ovine and Cervine Recombinant PrPs

A wide variety of TSE agents, including both classical and atypical BSE strains, as well as four scrapie strains and a CWD strain were used as seeding agents obtained from definitively diagnosed TSE brain tissues of cattle, sheep, and elk, respectively (Table 1). First, minute amounts of classical scrapie (Dawson, Langlade, UKA2 and SC21), BSE, atypical BSE (BSE-H and BSE-L), and CWD were subjected to serial rounds of rec-PMCA using ovine and cervine rec-PrP substrates complemented with *PRNP*^*−/−*^ brain homogenate. The PK-resistant PMCA products will be referred to as rec-PrP^res^ instead of PrP^Sc^ throughout this section as their infectivity in animal models has not been tested and, thus, cannot be referred to as PrP^Sc^ unless they are proven infectious in vivo, which is not the purpose of the present study.

**Fig. 1:**
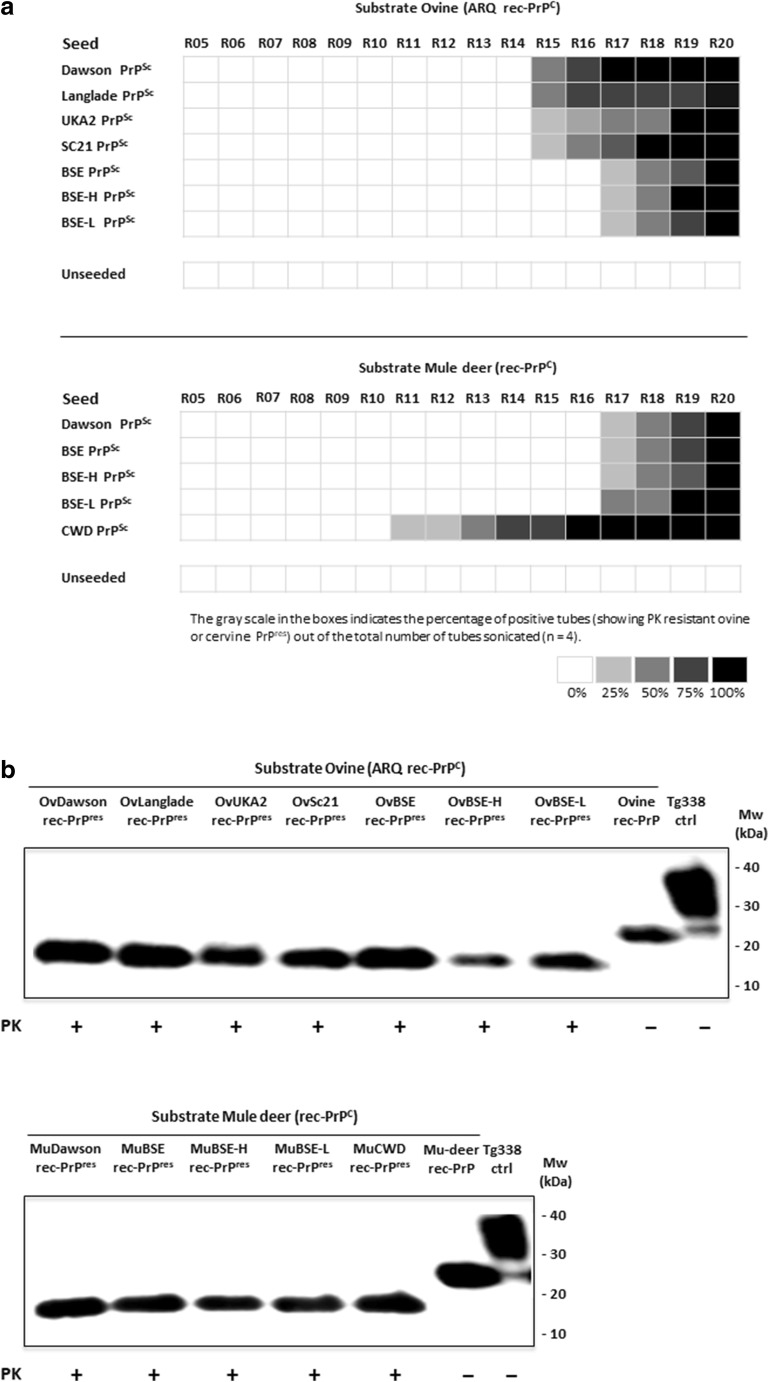
**a** Graphic representation of PrP^res^ showing tubes in rounds R05 to R20 of serial rec-PMCA using sheep and mule deer recombinant proteins as substrate. A substrate mixture consisting of ovine or cervine rec-PrP, complemented with *PRNP*^*−/−*^ brain homogenate and seeded with a panel of TSE agents, including cattle BSE, CWD, and different sheep scrapie prions (Dawson, Langlade, UKA2, Sc21) or unseeded were subjected to 20 rounds of rec-PMCA. The percentage of duplicate tubes showing PK-resistant misfolded rec-PrP seen in each round is indicated in a greyscale. **b** Western blot of different ovine rec-PrP^res^ (OvDawson rec-PrP^res^, OvLanglade rec-PrP^res^, OvUKA2 rec-PrP^res^, OvSc21 rec-PrP^res^, OvBSE rec-PrP^res^, OvBSE-H rec-PrP^res^, and OvBSE-L rec-PrP^res^) and cervine rec-PrP^res^ (MuDawson rec-PrP^res^, MuBSE rec-PrP^res^, MuBSE-H rec-PrP^res^, MuBSE-L rec-PrP^res^, and MuCWD rec-PrP^res^) generated in vitro after the 20th serial round of rec-PMCA using ovine and cervine recombinant proteins as substrates. The presence of rec-PrP^res^ was determined by subjecting the rec-PMCA product to 25 μg/ml PK digestion for 1 h at 42 °C, followed by Western blot analysis using 9A2 antibody (diluted 1:4000). Recombinant ovine or cervine PrPs (ovine rec-PrP and Mu-deer rec-PrP) and tg338 brain homogenate (Tg338 ctrl) were used as controls. PK, Proteinase K; MW, molecular weight

Neither ovine rec-PrP nor mule deer rec-PrP misfolded spontaneously in 20 serial rounds of PMCA, while ovine rec-PrP was misfolded by all sheep scrapie strains and BSE strains that were first detected at rounds 15 and 17, respectively (Fig. 1a). Similarly, CWD seeded mule deer rec-PrP misfolded rec-PrP appeared as early as the 11th round of PMCA. Conversely, mule deer rec-PrP seeded with scrapie (Dawson), BSE and atypical BSE (BSE-H, BSE-L), did not gave rise to misfolded cervine rec-PrP^res^ until round 17 as would be expected with an interspecies transmission barrier (Fig. 1a). For subsequent studies, a single PMCA tube from round 20 of each seeded ovine and cervid recombinant strains was selected and scaled up and all the rec-PrP^res^ generated from each strain used as seed were analyzed, quantified and rec-PrP^res^ amounts equalized (Fig. 1b). Both ovine and mule deer undigested rec-PrPs used as control (rec-ctrl) showed a one banded PrP pattern around 21 kDa similar to the non-glycosylated mammalian PrP^C^ band, whereas the misfolded and PK-treated ovine and cervine rec-PrP^res^ were 15 kDa bands (Fig. 1b).

### Biochemical Characterization of Rec-PrP^res^ by Evaluation of In Vitro Propagation Ability

**Fig. 2:**
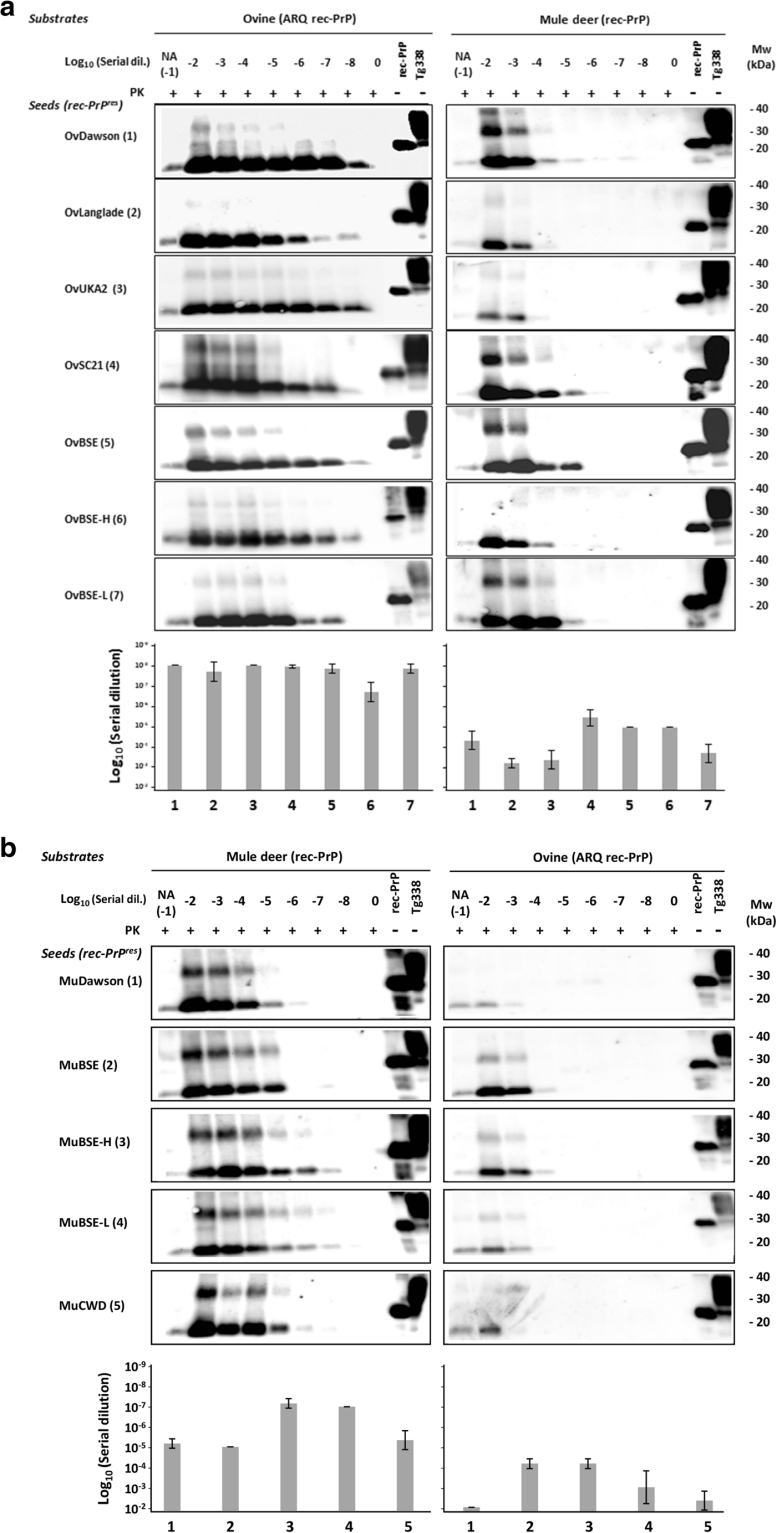
In vitro propagation of misfolded prion proteins. **a** Ovine (OvDawson rec-PrP^res^, OvLanglade rec-PrP^res^, OvUKA2 rec-PrP^res^, OvSc21 rec-PrP^res^, OvBSE rec-PrP^res^, OvBSE-H rec-PrP^res^, and OvBSE-L rec-PrP^res^) and **b** cervine (MuDawson rec-PrP^res^, MuBSE rec-PrP^res^, MuBSE-H rec-PrP^res^, MuBSE-L rec-PrP^res^, and MuCWD rec-PrP^res^) misfolded PK-resistant proteins were diluted from 10^−2^ to 10^−8^ and amplified by rec-PMCA using ovine ARQ rec-PrP and mule deer rec-PrP as substrates. The negative control samples (0) were either unseeded ovine ARQ rec-PrP or cervine rec-PrP substrates. NA indicates the no-amplification sample which was diluted 10^−1^ but not subjected to PMCA. The products of a single PMCA round (48 h) were digested with PK (25 μg/ml) for 1 h at 42 °C, analyzed by Western blot and developed with 9A2 antibody (diluted 1:4000). Recombinant ovine or cervine PrPs (rec-PrP) and tg338 brain homogenate (Tg338) were used as controls. PK, Proteinase K; MW, molecular weight. Below the panel of Western blots is a representative experiment from three replicates, maximum dilutions for each strain are plotted as an average of three independent experiments, including standard deviations

To ensure the various rec-PrP^res^ derived from serial rounds of PMCA were able to propagate their misfolded conformation and to characterize them biochemically, as they were the products of seeding with several different strains, each rec-PrP^res^ was diluted serially in substrates containing the same or heterologous rec-PrP as the seed to evaluate also their transmissibility across species. The dilutions were then subjected to a single 48 h round of PMCA and the propagation ability of each strain in each substrate was determined by PK digestion and Western blot. All ovine misfolded proteins (OvDawson rec-PrP^res^, OvLanglade rec-PrP^res^, OvUKA2 rec-PrP^res^, OvSc21 rec-PrP^res^, OvBSE rec-PrP^res^, OvBSE-H rec-PrP^res^, and OvBSE-L rec-PrP^res^) were able to propagate on their own substrate containing recombinant ovine PrP (ARQ) up to at least dilution 10^−7^ (Fig. 2a). However, the same inoculum propagated in heterologous substrate containing the recombinant mule deer PrP showed a variable and consistently much lower propagation ability as expected with the presence of an interspecies transmission barrier (Fig. 2a).

The cervine misfolded proteins (MuDawson rec-PrP^res^, MuBSE rec-PrP^res^, MuBSE-H rec-PrP^res^, MuBSE-L rec-PrP^res^, MuCWD rec-PrP^res^) were able to propagate their conformation consistently on the substrate containing the mule deer rec-PrP up to dilutions between 10^−5^ and 10^−7^, while its propagation ability was variable and decreased by several orders in the substrate containing sheep rec-PrP (ARQ), reaching maximal dilutions of 10^−3^ or 10^−4^ (Fig. 2b). Surprisingly, the misfolded proteins MuDawson rec-PrP^res^ and MuCWD rec-PrP^res^ showed a very low propagation ability in the ovine recombinant PrP substrate, indicating a significant transmission barrier.

### Introducing Certain Substitutions from the Deer Prion Protein into Ovine Prion Protein Alters the In Vitro Propagation Ability of Some Deer and Ovine Prions

**Fig. 3:**
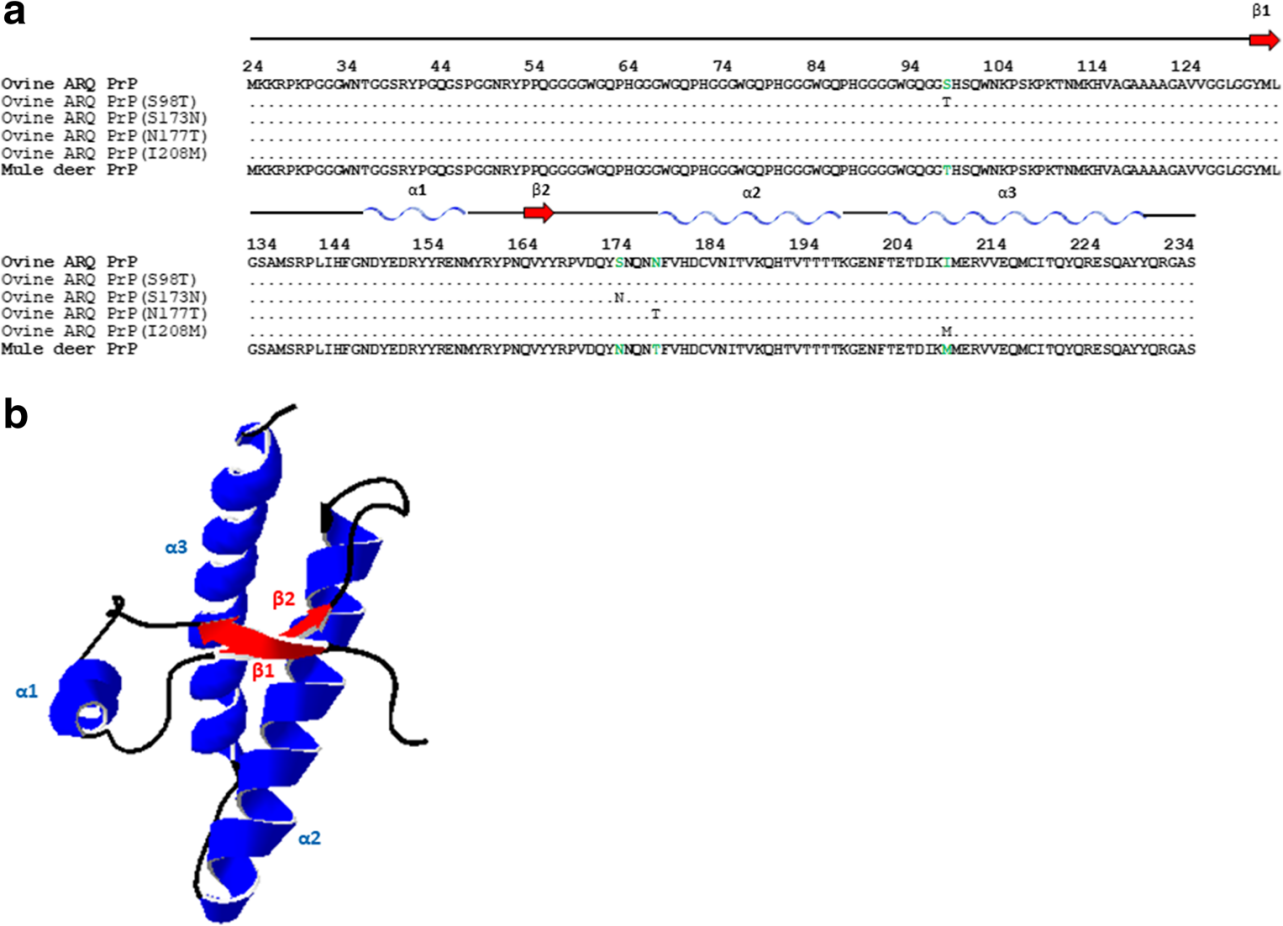
**a** Sequence alignment of the C-terminal domain (residues 134–234) of ovine PrP compared with mule deer PrP. Note the four amino acid differences that where substituted giving rise to four mutants: ovine ARQ PrP (S98T), ovine ARQ PrP (S173N), ovine ARQ PrP (N177T), and ovine ARQ PrP (I208M). **b** Diagram of ovine PrP, and the locations of the native secondary structures in sheep ARQ PrP (134–234) are indicated: the α-helical regions are represented in blue and the β-sheet region in red. The diagram was generated using the program Swiss-PdbViewer (4.1.0)

As shown previously by in vivo studies [14, 16], there is a stringent barrier against the transmission of cervine prions to ovine PrP protein and vice versa, which is mirrored by a significant decrease in the in vitro propagation abilities. To further investigate this transmission barrier phenomenon, an amino acid sequence comparison of both sheep and mule deer PrPs was undertaken and four amino acid residue differences were found, S98T, S173N, N177T and I208M (Fig. 3), which could be responsible for the observed transmission barrier.

To investigate the effect of each residue on the specie barrier and on the misfolding proneness of the ovine PrP, we performed a quantitative comparison of the propagation efficiency by serial dilution [10-2 to 10-8] of the recombinant inocula from the previous experiment in substrates containing ovine rec-PrP with individual S98T, S173N, N177T, and I208M substitutions and complemented with chicken brain homogenates. Resultant samples were then subjected to a single 48 h round of rec-PMCA with all the substrates adjusted previously to contain equal amounts of mutant and wild- type rec-PrPs.

**Fig. 4:**
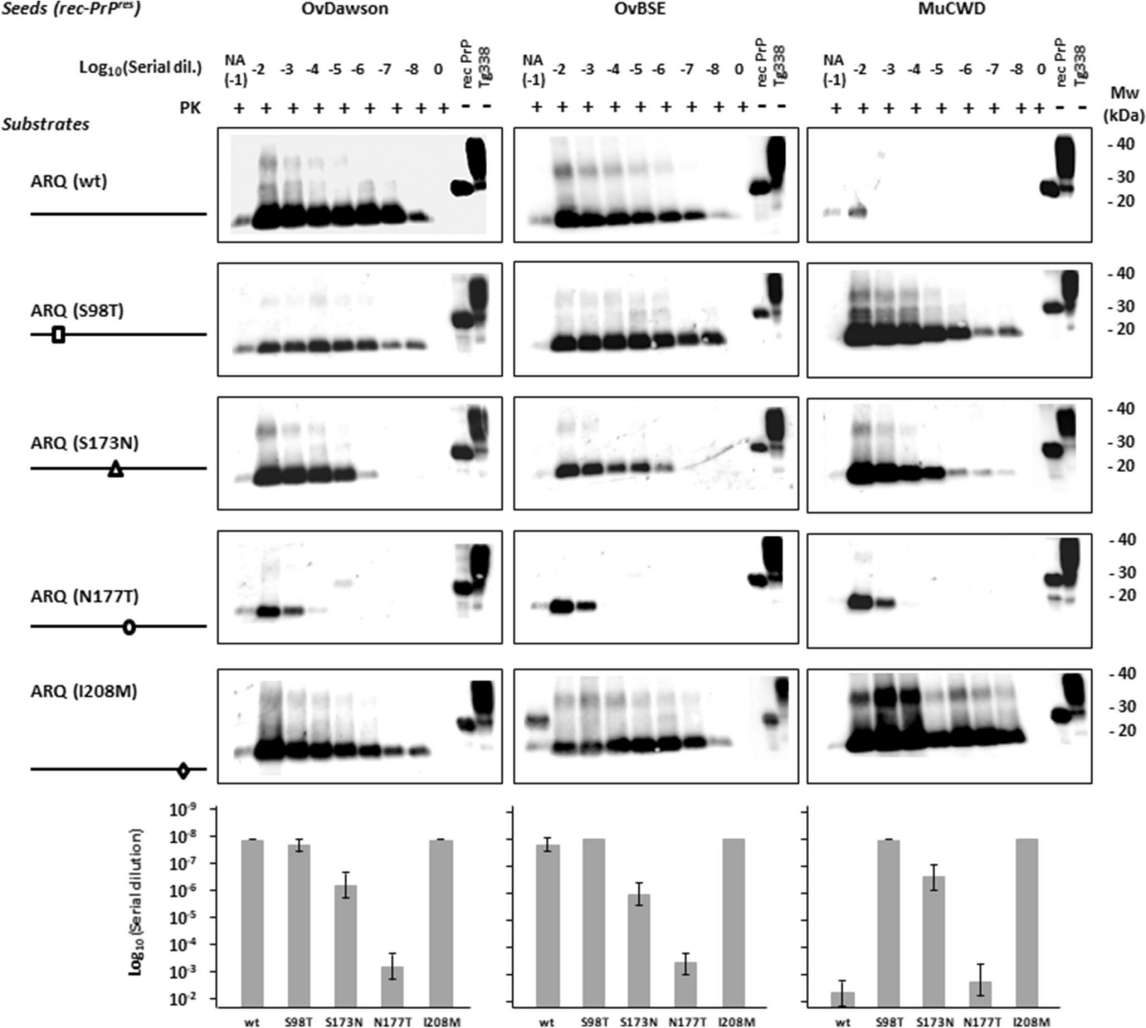
Evaluation of the effects of substitutions based on cervine PrP on the in vitro propagation of ovine (OvDawson rec-PrP^res^ and OvBSE rec-PrP^res^) and cervine (MuCWD rec-PrP^res^) misfolded proteins. Western blots showing PK-resistant PrP from 10^−2^ to 10^−8^ serial dilutions of OvDawson, OvBSE, and MuCWD rec-PrP^res^-seeded rec-PMCA propagation reactions containing wild-type (ovine ARQ), ARQ (S98T), ARQ (S173N), ARQ (N177T), and ARQ (I208M) rec-PrP substrates. For each seed, substrates containing ovine PrP substitutions were complemented with chicken brain homogenate and subjected to one round of rec-PMCA. The negative control samples (0) were either unseeded ovine ARQ rec-PrP or cervine rec-PrP substrate. NA indicates the no-amplification sample which was dilutes 10^−1^ but not subjected to PMCA. The products of a single PMCA round (48 h) were digested with PK (25 μg/ml) for 1 h at 42 °C, analyzed by Western blot, and developed with 9A2 antibody (diluted 1:4000). Recombinant ovine or cervine PrPs (rec-PrP) and tg338 brain homogenate (Tg338) were used as controls. In all blots, a sample containing wild-type ovine (ARQ rec-PrP) and Tg338 substrate not subjected to PK digestion is shown in the last lane. PK, Proteinase K; MW, molecular weight. Below the panel of Western blots is a representative experiment from three replicates, maximum dilutions for each strain are plotted as an average of three independent experiments, including standard deviations. The Western blots and results corresponding to N177T substitution are also presented in the next figure due to its relevant blocking effect in order to facilitate the interpretation of results in both figures

The ovine ARQ substrates showed the highest levels of propagation efficiency for ovine inocula OvDawson rec-PrP^res^ and OvBSE rec-PrP^res^. In contrast, no notable amplification was observed in the case of the cervine inoculum MuCWD rec-PrP^res^, using the same substrate (sheep ARQ PrP) (Fig. 4).

The S98T and I208M substitutions maintained the propagation efficiency seen with wild-type ovine ARQ-PrP^C^ for the ovine inocula OvDawson rec-PrP^res^ and OvBSE rec-PrP^res^, whereas a significant increase in replication ability was observed using the cervine inoculum MuCWD rec-PrP^res^ and the same substrates containing the S98T or I208M substitution compared to wild-type ovine rec-PrP, reaching 10^−8^ dilution in both cases. In contrast, the sheep substrate containing the S173N residue, despite being able to propagate MuCWD rec-PrP^res^ efficiently, had a reduced efficiency for OvDawson rec-PrP^res^ and OvBSE rec-PrP^res^ compared to the wild-type ovine PrP (Fig. 4). A decrease in amplification ability (4–5 log) was detected using the ovine substrate carrying the N177T mutant for the OvDawson and OvBSE rec-PrP^res^ but the CWD showed a similar lack of propagation ability as in the ovine wild-type substrate (Fig. 4).

### Introduction of Additional Mule Deer Substitutions into the Ovine Rec-PrP That Has Cervine Substitutions at the β2–α2 Loop Strongly Influences Interspecies Transmissibility In Vitro

Previous studies in both transgenic mouse and cell-culture models of prion conversion led to the identification of two residues within the loop between β-sheet 2 and α-helix 2 of the PrP which, when mutated, resulted in loop rigidity. This occurs in species such as the wallaby, horse, and rabbit and increases the propensity of the PrP to misfold in both in vitro and in vivo systems [28, 43–46]. Also, the important role of the rigid loop (β2–α2) on CWD PrP misfolding [47] warranted further exploration of its effects on ovine PrP. The rigidity in the β-sheet 2–α-helix 2 loop is provided by two amino acid substitutions in cervine PrP^C^ in comparison to other species: S173N and N177T [47]. To determine the effect of the β2–α2 loop on ovine prion protein misfolding and the effect of substitutions far from the loop on this critical region, some double and triple ovine PrP mutants were generated with cervine PrP substitutions and their misfolding ability was tested in vitro by serial dilution of the seeds.

**Fig. 5:**
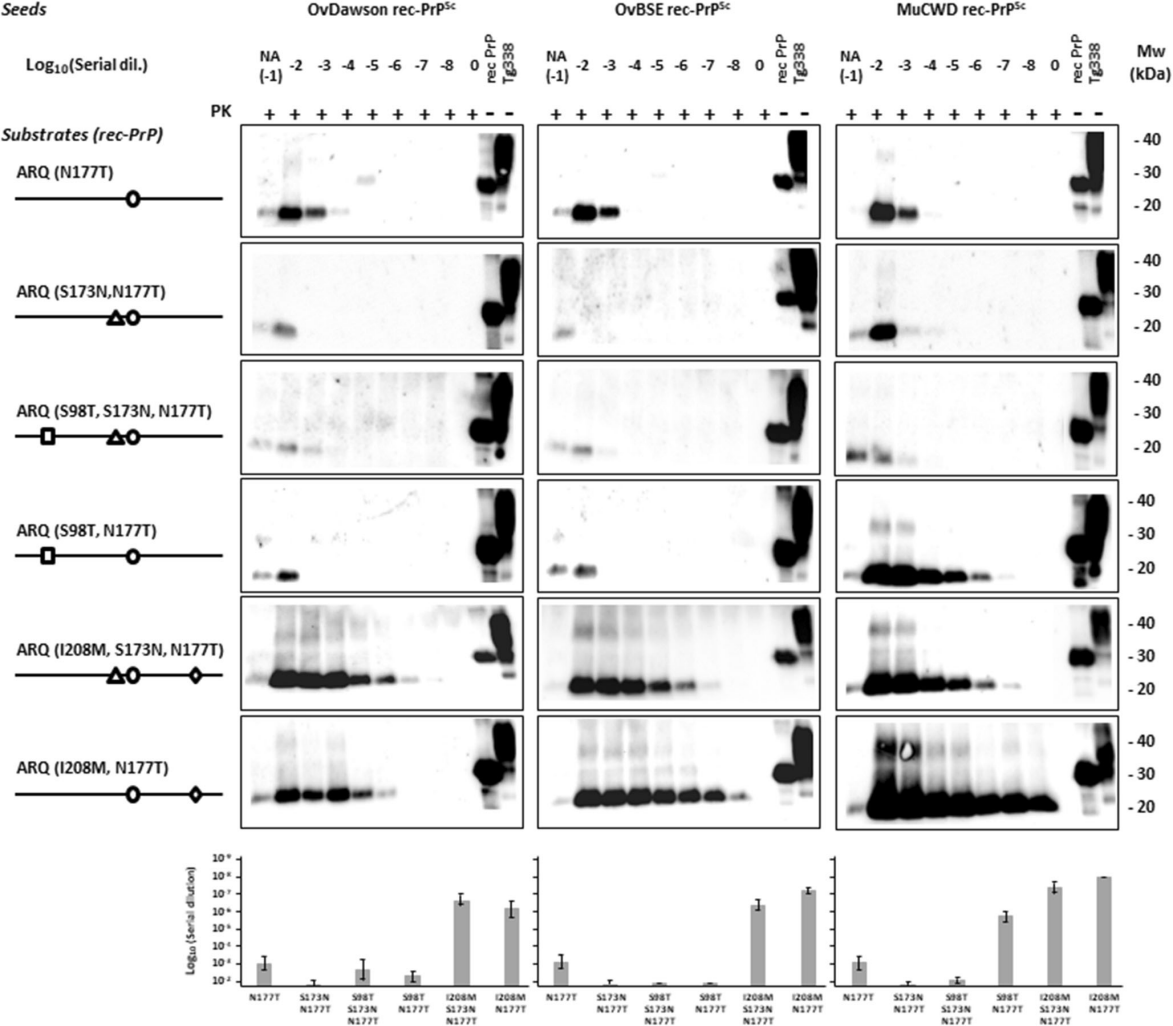
In vitro propagation ability of ovine and cervine recombinant misfolded seeds on substrates containing ovine rec-PrP with substitutions that define the cervine rigid loop (S173N T177N) and with other mule deer substitutions in combination with the ones defining the rigid loop. Western blots showing PK-resistant misfolded rec-PrP after serial dilutions from 10^−2^ to 10^−8^ of OvDawson, OvBSE, and MuCWD rec-PrP^res^ and a single 48 h rec-PMCA round on substrates containing ovine rec-PrP with substitutions (N177T), (S173N N177T), (S98T N177T), (I208M N177T), (S98T S173N N177T), and (I208M S173N N177T) complemented with chicken brain homogenate. The negative control samples (0) were unseeded ovine ARQ rec-PrP substrate. NA indicates the no-amplification sample with the inocula diluted 10^−1^ and not subjected to sonication. The rec-PMCA products were digested with PK (25 μg/ml) for 1 h at 42 °C, analyzed by Western blot, and probed with 9A2 antibody (diluted 1:4000). Recombinant ovine or cervine PrPs (rec-PrP) and tg338 brain homogenate (Tg338) were used as controls. PK, Proteinase K; MW, molecular weight. Below the panel of Western blots is a representative experiment of three replicates, and maximum dilutions for each strain are plotted as an average of three independent experiments, including standard deviations. The Western blots and results corresponding to N177T substitution are also presented in the previous figure due to its relevant blocking effect in order to facilitate the interpretation of results in both figures

The previous results highlighted that introduction of the N177T substitution into the ovine PrP sequence decreases its misfolding propensity in vitro when seeded with ovine and cervine misfolded proteins. The addition of S173N mutation to the N177T ovine rec-PrP (S173N, N177T), creating a rigid loop region identical in primary amino acid sequence to the cervine PrP, completely blocked its ability to propagate any of the tested seeds in vitro (Fig. 5). To determine whether the additional S98T substitution in ovine rec-PrP (S98T, S173N, N177T), which reduces the amino acid sequence differences between ovine and mule deer rec-PrP to a single residue, would recover ovine and cervine seed propagation, a single rec-PMCA round was performed with serial dilutions of ovine and cervine misfolded seeds on the ovine rec-PrP with the triple substitution (S98T, S173N, N177T) compared to the double mutants and the single mutant ovine rec-PrP with T177N substitution. The upper part of Fig. 5a shows that introduction of S98T substitution in the ovine rec-PrP with cervine rigid loop did not alter significantly the propagation ability of the ovine or cervine seeds. However, in the absence of one of the substitutions (S173N) that defines the β-sheet 2–α-helix 2 loop rigidity, S98T substitution together with the N177T showed a significant recovery of cervine seed propagation ability that was able to replicate until 10^−7^ dilution.

The same method was used to investigate the effect of the I208M substitution in ovine rec-PrP (I208M S173N T177N), the propagation ability of which was evaluated again by serial dilution of the seeds and a single rec-PMCA round (Fig. 5). Surprisingly, unlike the double rigid loop-containing ovine rec-PrP (S173N N177T), the triple mutant was highly permissive to misfolding by all the seeds used. Moreover, the double mutant containing just I208M and T177N substitutions was more permissive to misfolding, suggesting that I208M substitution is one of the major modulators of the interspecies transmission barrier between sheep and deer, at least in vitro.

## Discussion

One of the most intriguing phenomena related to prion biology is the existence of diverse strains exhibiting specific biological and biochemical characteristics [48]. Among those features, the ability of each strain to infect some species but not others stands out and is often referred to as the transmission barrier. The molecular basis of this process is still unknown but it is thought to be controlled, at least in part, by the differences in the primary PrP sequences of donor and recipient animals [17, 27, 49] that affect the structure and folding of the each prion protein [50]. Understanding the molecular mechanism behind transmission barriers and, thus, being able to predict possible new cross-species outbreaks has tremendous implications for public health, as highlighted by the transmission to humans of BSE by ingestion of bovine BSE infected tissues [51]. Indeed, one of the most alarming issues in prion disorders during the last decade has been the emergence and zoonotic potential of new strains originating, for example, in response to the implementation of selective breeding programs for the eradication of scrapie in sheep [52–54].

Despite scrapie being present in sheep for more than 200 years and consumed by human, transmission of scrapie to humans has never been reported. Thus, with the exception of a recent study showing transmission of scrapie to transgenic mice expressing human PrP [54], the zoonotic potential of scrapie has long been considered zero. On the contrary, BSE is widely accepted as the origin of the human variant CJD (vCJD) [51, 55, 56]. However, the existence of a few natural and some experimental cases of BSE in small ruminants [57–60] and its high experimental transmissibility to transgenic mice expressing human PrP raises concern about possible zoonoses from these species [61]. Unlike scrapie or BSE, the risk of transmission of CWD strains to humans remains poorly studied [62, 63] and cannot be ruled out at present, although the few experimental challenges of transgenic mice expressing human PrP with CWD always failed to achieve transmission [19, 21, 64–66]. However, the risk of transmission of a given prion strain from one species to another is not limited to direct transmission but may also occur through adaptation in an intermediate host species, e.g., CWD may transmit to human through an intermediate host such as a sheep as the latter has a PrP primary sequence that differs in only four amino acids to the native one in mule deer. For these reasons, we decided to investigate the transmission barrier between mule deer CWD and sheep to identify key amino acid residues that may influence it.

In vivo studies have shown previously the low transmission efficiency of the most abundant CWD strain to sheep. Intracerebral inoculations of Suffolk breed sheep with CWD resulted in incubation periods of around 2160 dpi, significantly longer than the 660 dpi mean incubation time for intracerebral challenge in mule deer [14, 67]. Similar transmission studies were recently performed in transgenic mice expressing either sheep (Tg338) or elk (TgElk) PrP. Inoculation of several classical scrapie prions into TgElk mice resulted in an attack rate of around 7% and no clinical disease at endpoint (389 dpi) in contrast to the 100% attack rate in Tg338 mice and incubation periods of 146 to 262 dpi depending on the strain, whereas inoculation of CWD yielded the opposite results with a 100% attack rate in TgElk mice and rapid onset of clinical disease (121 dpi) versus no clinical diseases or PrP^res^ accumulation in Tg338 mice at endpoint (500 dpi) [22]. Similarly, our findings showed that when CWD rec-PrP^res^ derived from the same strain was serially diluted in substrate containing ovine ARQ rec-PrP and subjected to rec-PMCA it is almost unable to propagate in vitro indicating the existence of a significant species transmission barrier.

Despite the similarity of both PrP sequences, there is a notable structural difference in the rigidity of the loop between the β-sheet 2 and the α-helix 2 on the cervine PrP (residues 165–175) compared to the flexibility of the loop in ovine PrP [47]. The difference in flexibility in this region could play a key role in interspecies transmission given its possible function as a species-specific surface recognition site for protein–protein interactions [68] and its relevance in the modulation of interspecies transmission of CWD was first observed using PMCA. Due to the high cost of performing in vivo CWD susceptibility studies on candidate species, Kurt and collaborators used PMCA to assess the transmissibility of this prion disease to 12 different mammalian species. Their result suggested that efficient conversion was more likely in species with Asp at position 173 (N173), as occurs in native cervine PrP, and it was impeded in 6 out of 7 species that have Ser at this position (S173) [69]. Another study evaluated the effect of the rigid loop when introduced in mouse PrP and clearly established the modulatory effect it has on disease susceptibility. A transgenic mouse model (TgMoPrP^RL^) with a rigid loop due to two amino acid substitutions (S170N and N174T, equivalent to 173 and 177 in cervine PrP numbering) in the PrP was generated. These mice had a high resistance to transmission of mouse prions as shown by prolonged incubation periods compared to wild-type controls and an increased susceptibility to that particular CWD strain [46]. However, the discovery of new CWD strains with strikingly distinct interspecies transmission properties, like the H95+ readily transmissible to mice [70], argues for a reduced effect of regions as the rigid loop on defining transmissibility. Further studies on this region through the generation of transgenic mice with a rigid loop obtained through a different substitution (D167S) suggested that the susceptibility to CWD is more dependent on the sequence similarity of the loops than conformation [71]. Nevertheless, the effect of this loop seems far more complex than a simple amino acid sequence similarity or difference and the relevance of local conformational changes was also clearly established in agreement with the results presented herein. A mouse PrP with S170N and N174T substitutions which resulted in a “rigid loop” was further modified by the addition of a Y169G substitution that causes a dramatic local conformational change of the rigid loop structure. The expression of this PrP in a transgenic mouse line made it resistant to different murine prion strains but, surprisingly unlike TgMoPrP^RL^, also to CWD prions [72]. Further studies on position 169 demonstrated that an aromatic amino acid residue in this position was required for proper rigid loop formation and recovery of the transmission barrier modulatory effects of positions 170 and 174 [73]. Moreover the generation of a series of transgenic mice expressing chimeric elk/mouse PrP to determine the key residues involved in CWD propagation highlighted, again, the importance of the rigid loop. Six of seven mice with S170N substitution (resulting in rigid β2–α2 loop) showed a 100% attack rate when challenged with CWD while the ones expressing the flexible mouse β2–α2 loop showed resistance to CWD regardless of other elk substitutions [74]. In addition to the influence on the transmission barrier between cervine and rodent prions, a similar study also showed the modulatory effect of the rigid loop between human and cervine prions. A transgenic mouse line expressing human PrP with four amino acid substitutions (M166V, E168Q, S170N, and N174T) that resulted in the expression of a human PrP with a cervine rigid loop structure showed prolonged incubation periods when challenged with human sporadic CJD but were susceptible to CWD infection [75].

Our findings strongly support these findings given that the N177T substitution in ovine PrP, analogous to mouse N174T, reduces the in vitro propagation of the ovine seeds used (Fig. 4). Moreover, the addition of the second S173N substitution that encodes the cervine rigid loop in ovine PrP increased the transmission barrier inhibiting completely the propagation of scrapie and ovine BSE (Fig. 5), although this substitution alone made ovine PrP permissive to all ovine and cervine seeds tested. Despite the fact that ovine PrP with the S173N substitution apparently abrogated the transmission barrier between the two species, surprisingly, the substitution N177T alone or together with S173N in the ovine PrP restricted the propagation of the ovine seeds as well as of CWD. This indicates that distinct elements within or far from the rigid loop exert different effects on the transmission barrier and that some amino acid residue matches might be as relevant as an overall similar local structure.

Substitutions in residues distant from the rigid loop also caused drastic changes in the transmission barrier. In fact, residues 98 and 208 alone have shown an important effect on the propagation of CWD on ovine PrP. The permissibility shown by both substitutions to the propagation of all the seeds tested reflects their importance on reducing the transmission barrier under study. Given the similar effect of both substitutions alone, the construction of a double mutant containing S98T and I208M was discarded and we focused on evaluating the effect of these substitutions together with those from the β2–α2 loop due to the importance of this region observed in previous studies. We thought it is possible that they could show such a dramatic effect through changes in the local structure of the β2–α2 loop as shown by the substitution S98T in presence of the substitutions in the loop that result in strikingly different propagation with all PrP^res^ seeds tested. Thus, it appears that each substitution can modulate the effect of the other, probably by their effects on the overall PrP structure or the local structure of the rigid loop region. Another single residue substitution located on α-helix 3 (I208M) which further increases the similarity between ovine and cervine PrP sequences, together with both rigid loop substitutions (S173N and N177T) also caused dramatic changes on the transmission barrier modulatory effect of the rigid loop substitutions. Indeed, the presence of methionine at position 208 increases the propensity of the ovine PrP with a cervid-like rigid loop to propagate all the seeds tested, including CWD (Fig. 5). Therefore, it is clear that other residues far from the β2–α2 loop are important determinants of the transmission barrier between cervine CWD and sheep, regardless their interactions with the rigid loop region.

This result, and the blocking of CWD propagation in ovine PrP substrate containing a rigid loop, suggests that for CWD propagation the presence of a cervine α-helix 3, through the I208M substitution, may be required to preserve the β2–α2 loop structural environment rather than complete sequence identity of the rigid loop. To test this hypothesis, the N177T I208M ovine PrP construct was generated where S173N, one of the mutations responsible for the formation of β2–α2 rigid loop, had been removed. We found that the presence of I208M allowed the propagation of all the seeds used, regardless of the presence or absence of S173N substitution, demonstrating the significant role of the I208M residue in PrP conversion compared to the role of the amino acid residue sequence identity of the β2–α2 loop.

Determination of the structures of mouse, hamster, and sheep PrP, among others, by NMR showed that the overall structure of mammalian PrPs is very similar. However, even a small number of amino acid changes can give raise to subtle structural variations that may have a dramatic effect on the susceptibility to misfolding by different prion strains [76]. Structural variations are particularly significant in the β2–α2 loop that was reported to be flexible in mouse PrP [77] while presenting a rigid structure in hamster PrP despite their overall structural similarity [78]. Previous studies on PrPs from several different species revealed a range of properties for this region, from completely flexible to highly rigid loops [79–81]. Although the rigidity in this loop generally correlates well with susceptibility to prion diseases, exceptions can be easily found [81, 82] showing that the regulation exerted by the rigid loop on misfolding susceptibility is much more complex. Rabbit PrP is one of the most interesting exceptions given that its structure, by X-ray spectroscopy, shows a well-defined rigid loop [83] despite this species having a very low susceptibility to prion infection [84–86]. Interestingly, the detailed structural study of rabbit PrP revealed local hydrogen bonds within the β2–α2 loop formed by the side chains of residues P165 and V166 with Q168 and Y169 [83]. Extensive structural analysis of PrPs from other species showed that although the global shape of the protein is highly conserved, amino acid substitutions within the β2–α2 loop dramatically alter the conformation of this region, modifying the hydrogen bonds and thus affecting the possible protein–protein interactions of this potentially species-dependent surface recognition site [68].

Our results show that alteration of position 173 in ovine PrP numbering is enough to change the susceptibility of ovine PrP to CWD propagation, probably by affecting similar local hydrogen bonds. Furthermore, the introduction of the second rigid loop encoding substitution, 177, seems to completely alter the arrangement of this region leading to an almost complete inhibition of the misfolding that could be due to a local arrangement more similar to that present in rabbit PrP. Additionally, both substitutions could destabilize the structure of ovine PrP to the extent of inhibiting its conversion into a protease-resistant form. This was initially suggested by studies showing that amino acid substitutions at codon 164 in the β2–α2 loop region of the mouse PrP protein produced local structural changes that may affect its global structure and stability, severely affecting PrP^C^ to PrP^res^ conversion in vitro [87]. Apparently, this structural destabilization could result from the disruption of salt bridges essential for the overall stability of the protein structure [88, 89].

However, even if disruption of a salt bridge could explain a complete block of PrP^res^ propagation due to the structural destabilization of the prion protein, this hypothesis is not in agreement with our findings that the propagation ability of our ovine PrP mutants is reduced but rarely completely inhibited. Moreover, it would be difficult to explain how, in the case of the cervine rigid loop-containing sheep PrP that only has two differences in its primary amino acid sequence compared to the mule deer PrP (positions 98 and 208), could have such a different local arrangement that it impedes CWD propagation. Thus, besides differences in the β2–α2 loop structure identity between donor and recipient species, some other region must influence the transmission barrier between ovine and cervine PrP. Some NMR studies on the structure of PrPs from species considered resistant to prion infection, such as horse and rabbit, have suggested that their low misfolding ability is determined not just by the presence of the rigid loop but by its interactions with the carboxi terminal of α-helix 3 [44, 90]. Remarkably, our results showed that an additional single amino acid substitution within α-helix 3 (I208M) in the ovine PrP with cervid β2–α2 loop was sufficient to remove the cervine-to-ovine transmission barrier almost completely, supporting the importance of the interactions between the β2–α2 loop and α-helix 3.

**Fig. 6:**
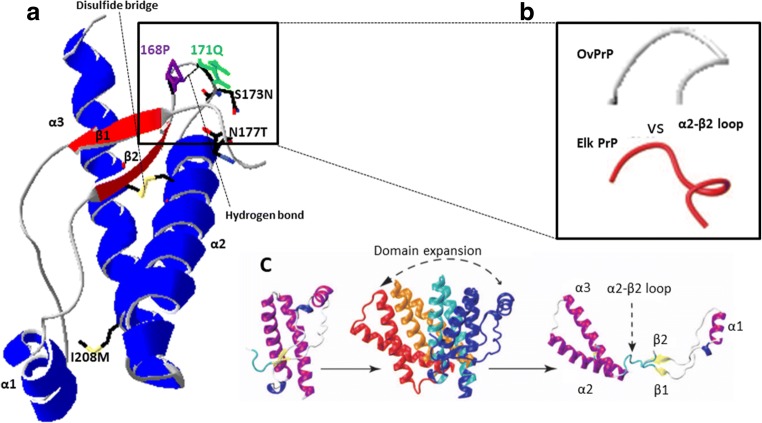
**a** Ribbon diagram of the C-terminal domain of ovine PrP-ARQ (residues 114–234, pdb 1TPX). α-helices in blue and β-sheets in red. Amino acid residues involved in the structural arrangement of β2–α2 loop and α-helix 3 (I208M) are indicated. Hydrogen bonds are shown as dashed black lines. The disulfide bridge is represented by the yellow connection between α-helix 2 and 3. The diagram was generated using the software Swiss-PdbViewer v4.1.0. **b** The flexible ovine β2–α2 loop (gray) versus the cervine rigid loop β2–α2 (red) (adapted from [47]). **c** Prion structural dynamics and mechanism of separation of β1–α1–β2 from α2-α3 in relation to the polymerization process (adapted from [91])

The relevance of the interaction between the β2–α2 loop and α-helix 3 was also established by Prigent and Rezaei by studying PrP oligomer formation in vitro. The role of these regions on the formation and conformational diversity of PrP oligomers generated by thermal treatment was demonstrated by increasing the rigidity of the loop through a disulfide bond between β2–α2 loop and helix α3 that significantly reduced the formation of oligomers [91]. Several studies have suggested that one of the early events leading to pathogenic oligomer formation consists of the physical expansion of the α2–α3 helix domain from the rest of the protein [91–98]. Consistent with the results from the previous studies and our own results, molecular dynamics simulations on ovine PrP have also shown that single amino acid residue mutations located in the α2 and α3 helices dramatically and selectively affect the PrP protein oligomerization process. In particular, the mutation I208M, localized in the α3 helix (Fig. 6a), led to the formation of slightly different oligomers than the wild-type PrP under specific pH and temperature conditions. Furthermore, molecular dynamics simulations showed that this protein refolds into a β-sheeted double hairpin, consisting of 2 β-hairpins linked by a loop, similar to the wild-type protein but with lower β-sheet content [99, 100, 101].

The data from all the above studies suggests the I208M substitution in ovine PrP could interact with the β2–α2 rigid loop altering the disulfide bridge cysteine residues at positions 182 and 218 [102]. The interaction between the 208M residue and the β2–α2 rigid loop region may induce a higher conformational instability of ovine PrP^C^ leading to the rupture of the disulfide bridge (Fig. 6a), causing the opening of the β2–α2 rigid loop, considered a hinge, and thus aiding the expansion of α1–β2 from the α2–α3 domain (Fig. 6c).

Collectively, these results suggest a significant role for position 208 and a possible interaction of the α3 helix with the β2–α2 loop that strongly influences not only the transmission of this CWD strain to sheep but also PrP^C^ conversion. These findings are in agreement with our results that show how inhibition of CWD propagation caused by introducing the β2–α2 rigid loop structure from deer into ovine PrP is abrogated by the presence of a methionine at position 208 of the ovine prion protein. Although a mechanistic explanation of this phenomenon will require further structural studies, our results provide further experimental support of the relevance of interactions between the α3 helix and the β2–α2 loop with respect to misfolding of PrP^C^. Moreover, our results establish that the rigid loop is not the only determinant of the interspecies transmission barrier between deer and sheep and that its effect might be modulated through complex interactions with the α3 helix.

## References

[1] Prusiner SB (1982). Novel proteinaceous infectious particles cause scrapie. Science.

[2] Detwiler LA, Baylis M (2003). The epidemiology of scrapie. Rev Sci Tech.

[3] Hunter AJ, Caulfield MP, Kimberlin RH (1986). Learning ability of mice infected with different strains of scrapie. Physiol Behav.

[4] Bessen RA, Kocisko DA, Raymond GJ, Nandan S, Lansbury PT, Caughey B (1995). Non-genetic propagation of strain-specific properties of scrapie prion protein. Nature.

[5] Williams ES, Young S (1980). Chronic wasting disease of captive mule deer: a spongiform encephalopathy. J Wildl Dis.

[6] Haley NJ, Hoover EA (2015). Chronic wasting disease of cervids: current knowledge and future perspectives. Annu Rev Anim Biosci.

[7] Sohn HJ, Kim JH, Choi KS, Nah JJ, Joo YS, Jean YH, Ahn SW, Kim OK, Kim DY, Balachandran A (2002). A case of chronic wasting disease in an elk imported to Korea from Canada. J Vet Med Sci.

[8] Benestad SL, Mitchell G, Simmons M, Ytrehus B, Vikoren T (2016). First case of chronic wasting disease in Europe in a Norwegian free-ranging reindeer. Vet Res.

[9] Benestad SL, Telling GC (2018). Chronic wasting disease: an evolving prion disease of cervids. Handb Clin Neurol.

[10] Williams ES, Young S (1992). Spongiform encephalopathies in Cervidae. Rev Sci Tech.

[11] Angers RC, Kang HE, Napier D, Browning S, Seward T, Mathiason C, Balachandran A, McKenzie D, Castilla J, Soto C, Jewell J, Graham C, Hoover EA, Telling GC (2010). Prion strain mutation determined by prion protein conformational compatibility and primary structure. Science.

[12] Bruce M, Chree A, McConnell I, Foster J, Pearson G, Fraser H (1994). Transmission of bovine spongiform encephalopathy and scrapie to mice: strain variation and the species barrier. Philos Trans R Soc Lond Ser B Biol Sci.

[13] Bartz JC, Marsh RF, McKenzie DI, Aiken JM (1998). The host range of chronic wasting disease is altered on passage in ferrets. Virology.

[14] Hamir AN, Kunkle RA, Cutlip RC, Miller JM, Williams ES, Richt JA (2006). Transmission of chronic wasting disease of mule deer to Suffolk sheep following intracerebral inoculation. J Vet Diagn Investig.

[15] Hamir AN, Kunkle RA, Richt JA, Miller JM, Cutlip RC, Jenny AL (2005). Experimental transmission of sheep scrapie by intracerebral and oral routes to genetically susceptible Suffolk sheep in the United States. J Vet Diagn Investig.

[16] Hamir AN, Miller JM, Cutlip RC, Stack MJ, Chaplin MJ, Jenny AL (2003). Preliminary observations on the experimental transmission of scrapie to elk (Cervus elaphus nelsoni) by intracerebral inoculation. Vet Pathol.

[17] Scott M, Groth D, Foster D, Torchia M, Yang SL, DeArmond SJ, Prusiner SB (1993). Propagation of prions with artificial properties in transgenic mice expressing chimeric PrP genes. Cell.

[18] Browning SR, Mason GL, Seward T, Green M, Eliason GA, Mathiason C, Miller MW, Williams ES, Hoover E, Telling GC (2004). Transmission of prions from mule deer and elk with chronic wasting disease to transgenic mice expressing cervid PrP. J Virol.

[19] Kong Q, Huang S, Zou W, Vanegas D, Wang M, Wu D, Yuan J, Zheng M, Bai H, Deng H, Chen K, Jenny AL, O'Rourke K, Belay ED, Schonberger LB, Petersen RB, Sy MS, Chen SG, Gambetti P (2005). Chronic wasting disease of elk: transmissibility to humans examined by transgenic mouse models. J Neurosci.

[20] LaFauci G, Carp RI, Meeker HC, Ye X, Kim JI, Natelli M, Cedeno M, Petersen RB, Kascsak R, Rubenstein R (2006). Passage of chronic wasting disease prion into transgenic mice expressing Rocky Mountain elk (Cervus elaphus nelsoni) PrPC. J Gen Virol.

[21] Tamguney G, Giles K, Bouzamondo-Bernstein E, Bosque PJ, Miller MW, Safar J, DeArmond SJ, Prusiner SB (2006). Transmission of elk and deer prions to transgenic mice. J Virol.

[22] Madsen-Bouterse SA, Schneider DA, Zhuang D, Dassanyake RP, Balachandran A, Mitchell GB, O'Rourke KI (2016). Primary transmission of CWD versus scrapie prions from small ruminants to ovine and cervid PrP transgenic mice. J Gen Virol.

[23] Green KM, Browning SR, Seward TS, Jewell JE, Ross DL, Green MA, Williams ES, Hoover EA, Telling GC (2008). The elk PRNP codon 132 polymorphism controls cervid and scrapie prion propagation. J Gen Virol.

[24] Tamguney G, Miller MW, Giles K, Lemus A, Glidden DV, DeArmond SJ, Prusiner SB (2009). Transmission of scrapie and sheep-passaged bovine spongiform encephalopathy prions to transgenic mice expressing elk prion protein. J Gen Virol.

[25] Duque Velasquez C, Kim C, Herbst A, Daude N, Garza MC, Wille H, Aiken J, McKenzie D (2015). Deer prion proteins modulate the emergence and adaptation of chronic wasting disease strains. J Virol.

[26] Hannaoui S, Amidian S, Cheng YC, Duque Velasquez C, Dorosh L, Law S, Telling G, Stepanova M, McKenzie D, Wille H, Gilch S (2017). Destabilizing polymorphism in cervid prion protein hydrophobic core determines prion conformation and conversion efficiency. PLoS Pathog.

[27] Scott M, Foster D, Mirenda C, Serban D, Coufal F, Walchli M, Torchia M, Groth D, Carlson G, DeArmond SJ, Westaway D, Prusiner SB (1989). Transgenic mice expressing hamster prion protein produce species-specific scrapie infectivity and amyloid plaques. Cell.

[28] Vorberg I, Groschup MH, Pfaff E, Priola SA (2003). Multiple amino acid residues within the rabbit prion protein inhibit formation of its abnormal isoform. J Virol.

[29] Watts JC, Giles K, Stohr J, Oehler A, Bhardwaj S, Grillo SK, Patel S, DeArmond SJ, Prusiner SB (2012). Spontaneous generation of rapidly transmissible prions in transgenic mice expressing wild-type bank vole prion protein. Proc Natl Acad Sci U S A.

[30] Kocisko DA, Priola SA, Raymond GJ, Chesebro B, Lansbury PT, Caughey B (1995). Species specificity in the cell-free conversion of prion protein to protease-resistant forms: a model for the scrapie species barrier. Proc Natl Acad Sci U S A.

[31] Lee LY, Chen RP (2007). Quantifying the sequence-dependent species barrier between hamster and mouse prions. J Am Chem Soc.

[32] Castilla J, Saa P, Morales R, Abid K, Maundrell K, Soto C (2006). Protein misfolding cyclic amplification for diagnosis and prion propagation studies. Methods Enzymol.

[33] Castilla J, Saa P, Hetz C, Soto C (2005). In vitro generation of infectious scrapie prions. Cell.

[34] Saa P, Castilla J, Soto C (2006). Ultra-efficient replication of infectious prions by automated protein misfolding cyclic amplification. J Biol Chem.

[35] Castilla J, Gonzalez-Romero D, Saa P, Morales R, De Castro J, Soto C (2008). Crossing the species barrier by PrP (Sc) replication in vitro generates unique infectious prions. Cell.

[36] Castilla J, Morales R, Saa P, Barria M, Gambetti P, Soto C (2008). Cell-free propagation of prion strains. EMBO J.

[37] Fernández-Borges N, Erana H, Elezgarai SR, Harrathi C, Venegas V, Castilla J, Lawson VA (2017). A quick method to evaluate the effect of the amino acid sequence in the misfolding proneness of the prion protein. Prions: methods and protocols.

[38] Elezgarai SR, Fernández-Borges N, Erana H, Sevillano A, Moreno J, Harrathi C, Saá P, Gil D, Kong Q, Requena JR, Andreoletti O, Castilla J (2017). Generation of a new infectious recombinant prion: a model to understand Gerstmann–Sträussler–Scheinker syndrome. Sci Rep.

[39] Fernández-Borges N, Di Bari MA, Erana H, Sanchez-Martin M, Pirisinu L, Parra B, Elezgarai SR, Vanni I, Lopez-Moreno R, Vaccari G, Venegas V, Charco JM, Gil D, Harrathi C, D'Agostino C, Agrimi U, Mayoral T, Requena JR, Nonno R, Castilla J (2017). Cofactors influence the biological properties of infectious recombinant prions. Acta Neuropathol.

[40] Wilham JM, Orru CD, Bessen RA, Atarashi R, Sano K, Race B, Meade-White KD, Taubner LM, Timmes A, Caughey B (2010). Rapid end-point quantitation of prion seeding activity with sensitivity comparable to bioassays. PLoS Pathog.

[41] Bueler H, Fischer M, Lang Y, Bluethmann H, Lipp HP, DeArmond SJ, Prusiner SB, Aguet M, Weissmann C (1992). Normal development and behaviour of mice lacking the neuronal cell-surface PrP protein. Nature.

[42] Castilla J, Gutierrez-Adan A, Brun A, Pintado B, Salguero FJ, Parra B, Segundo FD, Ramirez MA, Rabano A, Cano MJ, Torres JM (2005). Transgenic mice expressing bovine PrP with a four extra repeat octapeptide insert mutation show a spontaneous, non-transmissible, neurodegenerative disease and an expedited course of BSE infection. FEBS Lett.

[43] Christen B, Hornemann S, Damberger FF, Wuthrich K (2009). Prion protein NMR structure from tammar wallaby (Macropus eugenii) shows that the beta2-alpha2 loop is modulated by long-range sequence effects. J Mol Biol.

[44] Perez DR, Damberger FF, Wuthrich K (2010). Horse prion protein NMR structure and comparisons with related variants of the mouse prion protein. J Mol Biol.

[45] Zhang J (2009). Studies on the structural stability of rabbit prion probed by molecular dynamics simulations. J Biomol Struct Dyn.

[46] Sigurdson CJ, Nilsson KP, Hornemann S, Manco G, Fernandez-Borges N, Schwarz P, Castilla J, Wuthrich K, Aguzzi A (2010). A molecular switch controls interspecies prion disease transmission in mice. J Clin Invest.

[47] Gossert AD, Bonjour S, Lysek DA, Fiorito F, Wuthrich K (2005). Prion protein NMR structures of elk and of mouse/elk hybrids. Proc Natl Acad Sci U S A.

[48] Bruce ME, Boyle A, Cousens S, McConnell I, Foster J, Goldmann W, Fraser H (2002). Strain characterization of natural sheep scrapie and comparison with BSE. J Gen Virol.

[49] Hagiwara K, Hara H, Hanada K (2013). Species-barrier phenomenon in prion transmissibility from a viewpoint of protein science. J Biochem.

[50] Vanik DL, Surewicz KA, Surewicz WK (2004). Molecular basis of barriers for interspecies transmissibility of mammalian prions. Mol Cell.

[51] Will RG, Ironside JW, Zeidler M, Cousens SN, Estibeiro K, Alperovitch A, Poser S, Pocchiari M, Hofman A, Smith PG (1996). A new variant of Creutzfeldt-Jakob disease in the UK. Lancet.

[52] Hunter N (2003). Scrapie and experimental BSE in sheep. Br Med Bull.

[53] Buschmann A, Groschup MH (2005). TSE eradication in small ruminants—quo vadis?. Berl Munch Tierarztl Wochenschr.

[54] Cassard H, Torres JM, Lacroux C, Douet JY, Benestad SL, Lantier F, Lugan S, Lantier I, Costes P, Aron N, Reine F, Herzog L, Espinosa JC, Beringue V, Andreoletti O (2014). Evidence for zoonotic potential of ovine scrapie prions. Nat Commun.

[55] Bruce ME, Will RG, Ironside JW, McConnell I, Drummond D, Suttie A, McCardle L, Chree A, Hope J, Birkett C, Cousens S, Fraser H, Bostock CJ (1997). Transmissions to mice indicate that ‘new variant’ CJD is caused by the BSE agent. Nature.

[56] Hill AF, Desbruslais M, Joiner S, Sidle KC, Gowland I, Collinge J, Doey LJ, Lantos P (1997). The same prion strain causes vCJD and BSE. Nature.

[57] Foster JD, Hope J, Fraser H (1993). Transmission of bovine spongiform encephalopathy to sheep and goats. Vet Rec.

[58] Bellworthy SJ, Hawkins SA, Green RB, Blamire I, Dexter G, Dexter I, Lockey R, Jeffrey M, Ryder S, Berthelin-Baker C, Simmons MM (2005). Tissue distribution of bovine spongiform encephalopathy infectivity in Romney sheep up to the onset of clinical disease after oral challenge. Vet Rec.

[59] Eloit M, Adjou K, Coulpier M, Fontaine JJ, Hamel R, Lilin T, Messiaen S, Andreoletti O, Baron T, Bencsik A, Biacabe AG, Beringue V, Laude H, Le Dur A, Vilotte JL, Comoy E, Deslys JP, Grassi J, Simon S, Lantier F, Sarradin P (2005). BSE agent signatures in a goat. Vet Rec.

[60] Jeffrey M, Gonzalez L, Chong A, Foster J, Goldmann W, Hunter N, Martin S (2006). Ovine infection with the agents of scrapie (CH1641 isolate) and bovine spongiform encephalopathy: immunochemical similarities can be resolved by immunohistochemistry. J Comp Pathol.

[61] Plinston C, Hart P, Hunter N, Manson JC, Barron RM (2014). Increased susceptibility of transgenic mice expressing human PrP to experimental sheep bovine spongiform encephalopathy is not due to increased agent titre in sheep brain tissue. J Gen Virol.

[62] Williams ES (2005). Chronic wasting disease. Vet Pathol.

[63] Sigurdson CJ, Aguzzi A (2007). Chronic wasting disease. Biochim Biophys Acta.

[64] Sandberg MK, Al-Doujaily H, Sigurdson CJ, Glatzel M, O'Malley C, Powell C, Asante EA, Linehan JM, Brandner S, Wadsworth JD, Collinge J (2010). Chronic wasting disease prions are not transmissible to transgenic mice overexpressing human prion protein. J Gen Virol.

[65] Wilson R, Plinston C, Hunter N, Casalone C, Corona C, Tagliavini F, Suardi S, Ruggerone M, Moda F, Graziano S, Sbriccoli M, Cardone F, Pocchiari M, Ingrosso L, Baron T, Richt J, Andreoletti O, Simmons M, Lockey R, Manson JC, Barron RM (2012). Chronic wasting disease and atypical forms of bovine spongiform encephalopathy and scrapie are not transmissible to mice expressing wild-type levels of human prion protein. J Gen Virol.

[66] Race B, Meade-White KD, Phillips K, Striebel J, Race R, Chesebro B (2014). Chronic wasting disease agents in nonhuman primates. Emerg Infect Dis.

[67] Greenlee JJ, Smith JD, Kunkle RA (2011). White-tailed deer are susceptible to the agent of sheep scrapie by intracerebral inoculation. Vet Res.

[68] Billeter M, Riek R, Wider G, Hornemann S, Glockshuber R, Wuthrich K (1997). Prion protein NMR structure and species barrier for prion diseases. Proc Natl Acad Sci U S A.

[69] Kurt TD, Telling GC, Zabel MD, Hoover EA (2009). Trans-species amplification of PrP (CWD) and correlation with rigid loop 170N. Virology.

[70] Herbst A, Duque Velasquez C, Triscott E, Aiken J, Mckenzie D (2017). Chronic wasting disease prion strain emergence and host range expansion. Emerg Infect Dis.

[71] Bett C, Fernandez-Borges N, Kurt TD, Lucero M, Nilsson KP, Castilla J, Sigurdson CJ (2012). Structure of the beta2-alpha2 loop and interspecies prion transmission. FASEB J.

[72] Kurt TD, Bett C, Fernandez-Borges N, Joshi-Barr S, Hornemann S, Rulicke T, Castilla J, Wuthrich K, Aguzzi A, Sigurdson CJ (2014). Prion transmission prevented by modifying the beta2-alpha2 loop structure of host PrPC. J Neurosci.

[73] Kurt TD, Jiang L, Bett C, Eisenberg D, Sigurdson CJ (2014). A proposed mechanism for the promotion of prion conversion involving a strictly conserved tyrosine residue in the beta2-alpha2 loop of PrPC. J Biol Chem.

[74] Tamguney G, Giles K, Oehler A, Johnson NL, DeArmond SJ, Prusiner SB (2013). Chimeric elk/mouse prion proteins in transgenic mice. J Gen Virol.

[75] Kurt TD, Jiang L, Fernandez-Borges N, Bett C, Liu J, Yang T, Spraker TR, Castilla J, Eisenberg D, Kong Q, Sigurdson CJ (2015). Human prion protein sequence elements impede cross-species chronic wasting disease transmission. J Clin Invest.

[76] Sweeting B, Khan MQ, Chakrabartty A, Pai EF (2010). Structural factors underlying the species barrier and susceptibility to infection in prion disease. Biochem Cell Biol.

[77] Riek R, Hornemann S, Wider G, Billeter M, Glockshuber R, Wuthrich K (1996). NMR structure of the mouse prion protein domain PrP(121-231). Nature.

[78] Donne DG, Viles JH, Groth D, Mehlhorn I, James TL, Cohen FE, Prusiner SB, Wright PE, Dyson HJ (1997). Structure of the recombinant full-length hamster prion protein PrP(29-231): the N terminus is highly flexible. Proc Natl Acad Sci U S A.

[79] López-Garcia F, Zhan R, Riek R, Wüthrich K (2000). NMR structure of the bovine prion protein. Proc Natl Acad Sci U S A.

[80] Zahn R, Liu A, Luhrs T, Riek R, von Schroetter C, Lopez Garcia F, Billeter M, Calzolai L, Wider G, Wuthrich K (2000). NMR solution structure of the human prion protein. Proc Natl Acad Sci U S A.

[81] Lysek DA, Schorn C, Nivon LG, Esteve-Moya V, Christen B, Calzolai L, von Schroetter C, Fiorito F, Herrmann T, Guntert P, Wuthrich K (2005). Prion protein NMR structures of cats, dogs, pigs, and sheep. Proc Natl Acad Sci U S A.

[82] Li J, Mei FH, Xiao GF, Guo CY, Lin DH (2007). 1H, 13C and 15N resonance assignments of rabbit prion protein (91-228). J Biomol NMR.

[83] Khan MQ, Sweeting B, Mulligan VK, Arslan PE, Cashman NR, Pai EF, Chakrabartty A (2010). Prion disease susceptibility is affected by beta-structure folding propensity and local side-chain interactions in PrP. Proc Natl Acad Sci U S A.

[84] Chianini F, Fernandez-Borges N, Vidal E, Gibbard L, Pintado B, de Castro J, Priola SA, Hamilton S, Eaton SL, Finlayson J, Pang Y, Steele P, Reid HW, Dagleish MP, Castilla J (2012). Rabbits are not resistant to prion infection. Proc Natl Acad Sci U S A.

[85] Vidal E, Fernández-Borges N, Pintado B, Eraña H, Ordoñez M, Márquez M, Chianini F, Fondevila D, Sánchez-Martín MA, Andreoletti O, Dagleish MP, Pumarola M, Castilla J (2015). Transgenic mouse bioassay: evidence that rabbits are susceptible to a variety of prion isolates. PLoS Pathog.

[86] Erana H**,** Fernandez-Borges N**,** Elezgarai SR**,** Harrathi C**,** Charco JM**,** Chianini F**,** Dagleish MP**,** Ortega G**,** Millet O**,** Castilla J**.** 2017. In vitro approach to identify key amino acids in low susceptibility of rabbit prion protein to misfolding. J Virol 91(24). 10.1128/JVI.01543-17 10.1128/JVI.01543-17 PMC5709604 28978705

[87] Kirby L, Agarwal S, Graham JF, Goldmann W, Gill AC (2010). Inverse correlation of thermal lability and conversion efficiency for five prion protein polymorphic variants. Biochemistry.

[88] Gill AC, Agarwal S, Pinheiro TJ, Graham JF (2010). Structural requirements for efficient prion protein conversion: cofactors may promote a conversion-competent structure for PrP(C). Prion.

[89] Guest WC, Cashman NR, Plotkin SS (2010). Electrostatics in the stability and misfolding of the prion protein: salt bridges, self energy, and solvation. Biochem Cell Biol.

[90] Wen Y, Li J, Xiong M, Peng Y, Yao W, Hong J, Lin D (2010). Solution structure and dynamics of the I214V mutant of the rabbit prion protein. PLoS One.

[91] Prigent S, Rezaei H (2011). PrP assemblies: spotting the responsible regions in prion propagation. Prion.

[92] Dima RI, Thirumalai D (2004). Probing the instabilities in the dynamics of helical fragments from mouse PrPC. Proc Natl Acad Sci U S A.

[93] Eghiaian F, Daubenfeld T, Quenet Y, van Audenhaege M, Bouin AP, van der Rest G, Grosclaude J, Rezaei H (2007). Diversity in prion protein oligomerization pathways results from domain expansion as revealed by hydrogen/deuterium exchange and disulfide linkage. Proc Natl Acad Sci U S A.

[94] Hafner-Bratkovic I, Gaedtke L, Ondracka A, Veranic P, Vorberg I, Jerala R (2011). Effect of hydrophobic mutations in the H2-H3 subdomain of prion protein on stability and conversion in vitro and in vivo. PLoS One.

[95] Xu Z, Adrover M, Pastore A, Prigent S, Mouthon F, Comoy E, Rezaei H, Deslys JP (2011). Mechanistic insights into cellular alteration of prion by poly-D-lysine: the role of H2H3 domain. FASEB J.

[96] Biljan I, Ilc G, Giachin G, Raspadori A, Zhukov I, Plavec J, Legname G (2011). Toward the molecular basis of inherited prion diseases: NMR structure of the human prion protein with V210I mutation. J Mol Biol.

[97] Biljan I, Giachin G, Ilc G, Zhukov I, Plavec J, Legname G (2012). Structural basis for the protective effect of the human prion protein carrying the dominant-negative E219K polymorphism. Biochem J.

[98] Hafner-Bratkovic I, Jerala R (2011). Disulfide mapping reveals the domain swapping as the crucial process of the structural conversion of prion protein. Prion.

[99] Chakroun N, Fornili A, Prigent S, Kleinjung J, Dreiss CA, Rezaei H, Fraternali F (2013). Decrypting prion protein conversion into a beta-rich conformer by molecular dynamics. J Chem Theory Comput.

[100] Chakroun N, Prigent S, Dreiss CA, Noinville S, Chapuis C, Fraternali F, Rezaei H (2010). The oligomerization properties of prion protein are restricted to the H2H3 domain. FASEB J.

[101] Gill AC (2014). β-Hairpin-mediated formation of structurally distinct multimers of neurotoxic prion peptides. PLoS One.

[102] Maiti NR, Surewicz WK (2001). The role of disulfide bridge in the folding and stability of the recombinant human prion protein. J Biol Chem.

